# Breast cancer and incident cardiovascular events: A systematic analysis at the nationwide level

**DOI:** 10.1111/eci.13754

**Published:** 2022-02-10

**Authors:** Ying X. Gue, Arnaud Bisson, Alexandre Bodin, Julien Herbert, Gregory Y. H. Lip, Laurent Fauchier

**Affiliations:** ^1^ Liverpool Centre for Cardiovascular Science University of Liverpool and Liverpool Heart & Chest Hospital Liverpool UK; ^2^ Service de Cardiologie Centre Hospitalier Universitaire et Faculté de Médecine Université de Tours Tours France; ^3^ Service d'information médicale, d'épidémiologie et d'économie de la santé Centre Hospitalier Universitaire et Faculté de Médecine Université de Tours Tours France

**Keywords:** breast cancer, cardiovascular death, stroke

## Abstract

**Background:**

Breast cancer (BC) is one of the most common cancers worldwide, and the treatments are frequently cardiotoxic. Whether BC is associated with a higher risk of cardiovascular events is a matter of debate. We evaluated the associations among BC and incident cardiovascular events in a contemporary population.

**Methods:**

All female patients discharged from French hospitals in 2013 with at least 5 years of follow‐up and without a history of major adverse cardiovascular event (myocardial infarction [MI], heart failure [HF], ischaemic stroke or all‐cause death, and MACE‐HF, which includes cardiovascular death, MI, ischaemic stroke or HF) or cancer (except BC) were identified. After propensity score matching, patients with BC were matched 1:1 with patients with no BC. Hazard ratios (HRs) for cardiovascular events during follow‐up were adjusted on age, sex and smoking status at baseline.

**Results:**

1,795,759 patients were included, among whom 64,480 (4.3%) had history of BC. During a mean follow‐up of 5.1 years, matched female patients with BC had a higher risk of all‐cause death (HR 3.55, 95% confidence interval [CI]: 3.47–3.64), new‐onset HF (HR 1.08, 95% CI 1.04–1.11), major bleeding (HR 1.43, 95% CI 1.36–1.49), MACE‐HF (HR 1.07, 95% CI 1.04–1.11) and net adverse clinical events (NACE) including all‐cause death, MI, ischaemic stroke, HF or major bleeding (HR 2.53, 95% CI 2.48–2.58) compared with those with no BC. By contrast, risks were not higher for cardiovascular death (HR 0.94, 95% CI 0.88–1.00) and were lower for MI (HR 0.81, 95% CI 0.75–0.88) and ischaemic stroke (HR 0.85, 95% CI 0.79–1.11).

**Conclusions:**

In a large and contemporary analysis of female patients seen in French hospitals, women with history of breast cancer had a higher risk of all‐cause mortality, new‐onset heart failure and major bleeding compared to a matched cohort of women without breast cancer. In contrast, they have a reduced risk of cardiovascular mortality, MI and stroke.

## 1 INTRODUCTION

Breast cancer (BC) is one of the most common cancers in women worldwide.^1^ The advancement in treatment for BC has improved tremendously through the years, and as a result of early detection and effective treatments, we have an increasingly larger cohort of BC survivors.^2^, ^3^

The increase in years of survival post‐diagnosis inevitably increases the prevalence of cardiovascular diseases (CVD) and deaths from cardiovascular causes in this group of patients. Not only is the increase in the risk of developing CVD attributed to the natural process of ageing, some evidence has also shown that cardiotoxic treatments such as mediastinal radiotherapy, anthracycline‐based chemotherapy, biological therapies and hormonal therapy for BC could also put these women at an increased risk, especially those with pre‐existing CVD risk factors.^4^, ^5^, ^6^

Anthracyclines, such as doxorubicin, remains a mainstay chemotherapeutic agent for patients with BC, in particular patients with low cardiovascular risk,^7^ as it has been shown to result in cardiotoxicity in the form of left ventricular dysfunction.^8^, ^9^ In patients with receptor‐positive BC, the use of endocrine therapy and human epidermal growth factor receptor 2 (HER2) blockade is recommended in conjunction with chemotherapy. In premenopausal women, ovarian suppression in combination with an aromatase inhibitor is commonly used as adjuvant endocrine therapy particularly in high‐risk patients who have received chemotherapy.^7^ Ovarian suppression with goserelin combined with an aromatase inhibitor (AI) results in profound suppression of oestradiol.^10^ Clinical studies of goserelin in combination with an AI have demonstrated several adverse metabolic effects, including glucose intolerance^11^ and hyperglycaemia, as well as hypertension, which can accelerate atherosclerosis and development of cardiovascular and cerebrovascular diseases.^12^, ^13^, ^14^

However, the interaction between these factors resulting in an increased risk of CVD and death from cardiovascular causes remains controversial. Studies utilising data from older databases such as those reported by Bradshaw et al. investigating a population of BC patients diagnosed in 1996 and 1997 showed an increased risk of CVD‐related death in BC survivors when compared to an age‐matched cohort of women without BC.^15^ On the contrary, Buddeke et al. investigating a larger time period, which included a more contemporary cohort (from 1995 to 2015), reported a *decrease* in the risk of cardiovascular mortality in patients with BC when compared to the general population.^16^ The stark difference in the conclusions drawn from these studies may be attributed to the heterogeneity of the patient population with different stages of BC, different background and, more importantly, the difference in monitoring and treatment of this cohort of patients.

Our aim was to evaluate the associations among BC and incident cardiovascular events in a contemporary population of female patients.

## 2 METHODS

Utilising the national hospitalisation database covering hospital care from the entire French population, we performed a retrospective longitudinal cohort study where the data for all patients admitted in French hospitals from January to December 2013 with at least 5 years of complete follow‐up (or earlier if death) were collected from the national administrative PMSI (Programme de Médicalisation des Systèmes d’Information) database, as previously described.^17^, ^18^, ^19^, ^20^ The reliability of PMSI data has already been assessed, and this database has previously been used to study patients with cardiovascular conditions.^17^, ^18^, ^19^, ^20^

As patients were not involved in its conduct, there was no impact on their care. As all data were anonymised, ethics approval specific to this study was not required. The French Data Protection Authority granted access to the PMSI data. Procedures for data collection and management were approved by the Commission Nationale de l'Informatique et des Libertés (CNIL), the independent National Ethical Committee protecting human rights in France, which ensures that all information is kept confidential and anonymous, in compliance with the Declaration of Helsinki (authorisation number 1897139).

### 2.1 Study population

From 1 January 2013 to 31 December 2013, 1,795,759 women (age ≥18 years) were hospitalised and had at least 5 years of complete follow‐up (or earlier if suffered death). Patient information such as demographics, past medical history and events during hospitalisation or follow‐up was described using data collected in the hospital records. For each hospital stay, combined diagnoses at discharge were obtained. Each variable was identified using International Classification of Diseases, Tenth Revision (ICD‐10) codes, and BC was identified with its ICD‐10 codes (C50 and its subsections). Exclusion criteria were age <18 years.

### 2.2 Outcomes

Patients were followed up until 31 December 2019 to identify any occurrence of events. We evaluated the incidence of all‐cause death, major cardiovascular events (MACE‐HF =cardiovascular death, myocardial infarction [MI], ischaemic stroke or new‐onset heart failure [HF]) and net adverse clinical events (NACE), which includes all‐cause death, MI, ischaemic stroke or new‐onset HF or major bleeding.

The endpoints were evaluated utilising follow‐up data starting from the date of first hospitalisation until the date of each specified outcome or date of last news in the absence of the outcome. Information on outcomes during the follow‐up was obtained by analysing the PMSI codes for each patient. Outcomes were identified using their respective ICD‐10, and the mode of death (cardiovascular or noncardiovascular) was identified based on the main diagnosis during hospitalisation resulting in death.

### 2.3 Statistical analysis

Baseline characteristics that are binary variables are described as frequency and percentages and continuous variable as means (standard deviations [SDs]). Multivariate analyses were performed using a Cox model with all baseline characteristics, and hazard ratio (HR) was reported. The model by Fine and Gray was also used for competing risks for (1) cardiovascular and noncardiovascular death, (2) MI and all‐cause death and (3) ischaemic stroke and all‐cause death.

As the study is nonrandomised and to account for the presence of significant differences in baseline characteristics and control for potential confounders, propensity score matching was performed. Propensity scores were calculated using logistic regression with BC as the dependent variable. The propensity score included cardiovascular risk factors and other comorbidities from the baseline characteristics listed in Table 1, namely age, sex, obesity, history of hypertension, diabetes, dyslipidaemia, smoking and alcohol‐related diagnoses.

**TABLE 1:** Baseline characteristics of women seen in French hospitals in 2013 with no history of cancer or history of breast cancer

	No breast cancer	Breast cancer	*p*	Total
	(n = 1424286)	(n = 64480)		(n = 1488766)
Age, years	53.8 ± 22.5	63.0 ± 13.7	<0.0001	54.2 ± 22.3
Sex (male)	0 (0.0)	0 (0.0)	‐	0 (0.0)
Obesity	145301 (10.2)	6484 (10.1)	0.23	151785 (10.2)
Hypertension	306368 (21.5)	17834 (27.7)	<0.0001	324202 (21.8)
Diabetes mellitus	145813 (10.2)	5873 (9.1)	<0.0001	151686 (10.2)
Dyslipidaemia	116058 (8.1)	6668 (10.3)	<0.0001	122726 (8.2)
Smoker	60328 (4.2)	3027 (4.7)	<0.0001	63355 (4.3)
Alcohol‐related diagnoses	36541 (2.6)	1318 (2.0)	<0.0001	37859 (2.5)
Valve disease	20660 (1.5)	1032 (1.6)	0.002	21692 (1.5)
Coronary artery disease	38395 (2.7)	1532 (2.4)	<0.0001	39927 (2.7)
Previous PCI	5918 (0.4)	165 (0.3)	<0.0001	6083 (0.4)
Previous CABG	451 (0.0)	23 (0.0)	0.58	474 (0.0)
Vascular disease	37138 (2.6)	1290 (2.0)	<0.0001	38428 (2.6)
Atrial fibrillation	55780 (3.9)	2586 (4.0)	0.23	58366 (3.9)
Previous pacemaker or ICD	14985 (1.1)	607 (0.9)	0.01	15592 (1.0)
Intracranial bleeding	9859 (0.7)	316 (0.5)	<0.0001	10175 (0.7)
Chronic kidney disease	24257 (1.7)	827 (1.3)	<0.0001	25084 (1.7)
Lung disease	87393 (6.1)	3983 (6.2)	0.67	91376 (6.1)
Sleep apnoea syndrome	34292 (2.4)	1115 (1.7)	<0.0001	35407 (2.4)
Liver disease	28047 (2.0)	1424 (2.2)	<0.0001	29471 (2.0)
Thyroid diseases	91199 (6.4)	5273 (8.2)	<0.0001	96472 (6.5)
Inflammatory disease	72738 (5.1)	2048 (3.2)	<0.0001	74786 (5.0)
Anaemia	75684 (5.3)	6445 (10.0)	<0.0001	82129 (5.5)
Previous cancer	0 (0.0)	64480 (100.0)	‐	64480 (4.3)
Cognitive impairment	39629 (2.8)	1206 (1.9)	<0.0001	40835 (2.7)
Illicit drug use	3538 (0.2)	84 (0.1)	<0.0001	3622 (0.2)

Note

Values are n (%) or mean ± SD.

Mean follow‐up: 5.1 ± 1.3 years; median: 5.5; IQR: 5.2–5.8 years.

Abbreviations: CABG, coronary artery bypass graft; ICD, implantable cardioverter–defibrillator; IQR, interquartile range; PCI, percutaneous coronary intervention; SD, standard deviation.

For every female patient with BC, a propensity score–matched female patient with no BC was identified and selected with the one‐to‐one nearest neighbour method (with a calliper of 0.01 of the SD of the propensity score on the logit scale) and no replacement. The distributions of demographic data and comorbidities in the two cohorts were assessed with standardised differences, which were calculated as the difference in the means or proportions of a variable divided by a pooled estimate of the SD of that variable with 5% or less indicating a negligible difference between the means of the two cohorts (Figure S1 and Figure S2).

Statistical significance was taken at *p* < 0.05. All analyses were performed using Enterprise Guide 7.1 (SAS Institute Inc., SAS Campus Drive, Cary, North Carolina, USA) and STATA version 16.0 (Stata Corp, College Station, TX).

## 3 RESULTS

**FIGURE 1:**
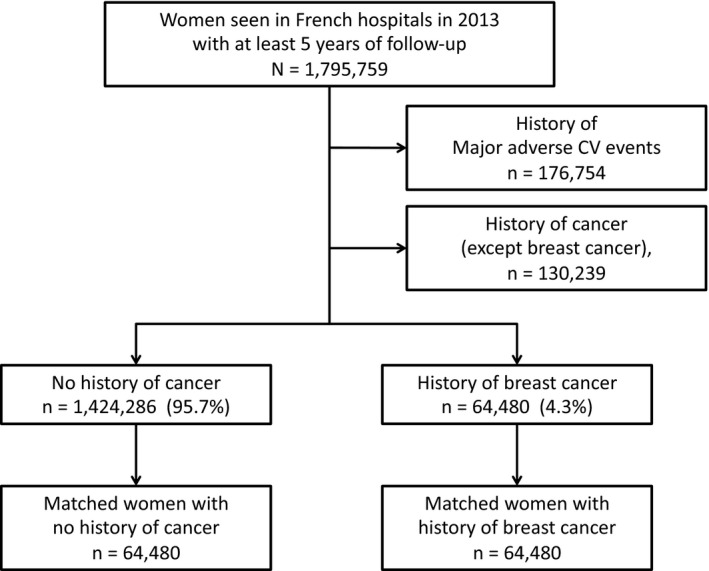
Flow chart of the study population

Among 1,795,759 female patients seen in French hospitals in 2013 with no history of MACE‐HF, 64,480 (4.3%) had BC (Table 1 and Figure 1). Comparison of baseline characteristics showed that female patients with history of BC were older and had a higher proportion of risk factors and comorbidities than those without BC.

During a mean follow‐up of 5.1 ± 1.3 years (median: 5.5, IQR: 5.2–5.8 years), 212,163 (11.8%) female patients with new‐onset MACE‐HF events were identified, which included 27,223 (1.5%) patients with MI, 38,271 (2.1%) patients with ischaemic stroke, 160,985 (9.0%) patients with HF and 46,943 (2.6%) patients with cardiovascular deaths. There were 74,257 (4.1%) female patients with major bleeding during follow‐up, and all‐cause death occurred in 234,303 (13%) female patients, resulting in 345,760 (19.3%) female patients with NACE.

**FIGURE 2:**
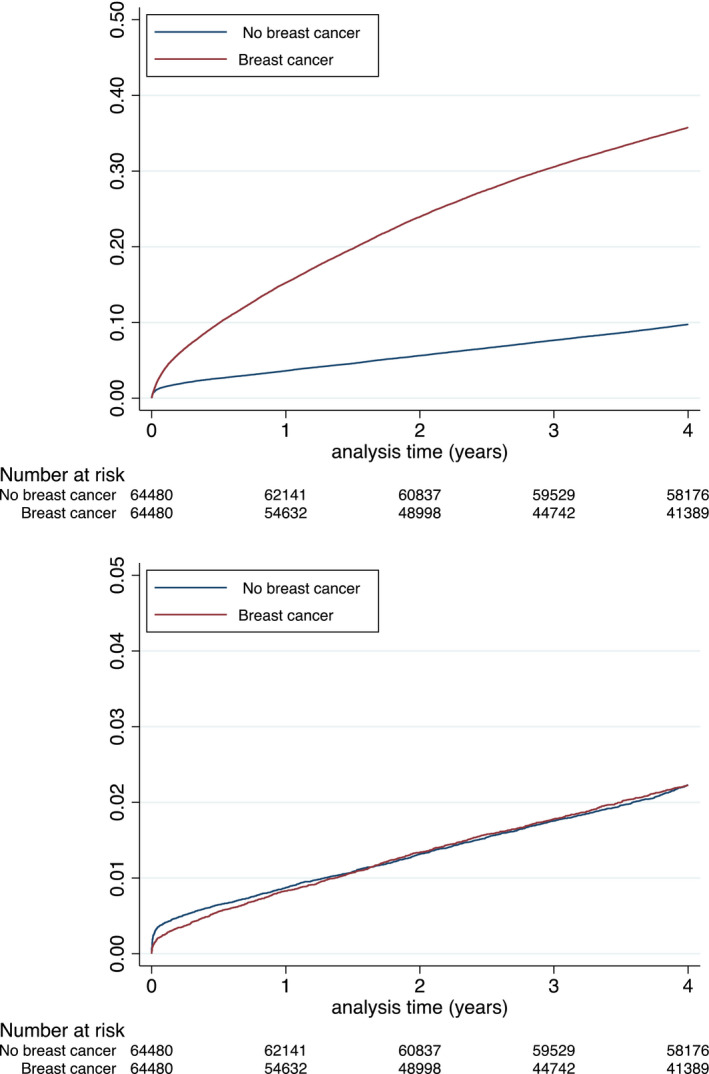
Cumulative incidences for all‐cause death (top panel) or cardiovascular death (lower panel) during follow‐up in the matched populations

**FIGURE 3:**
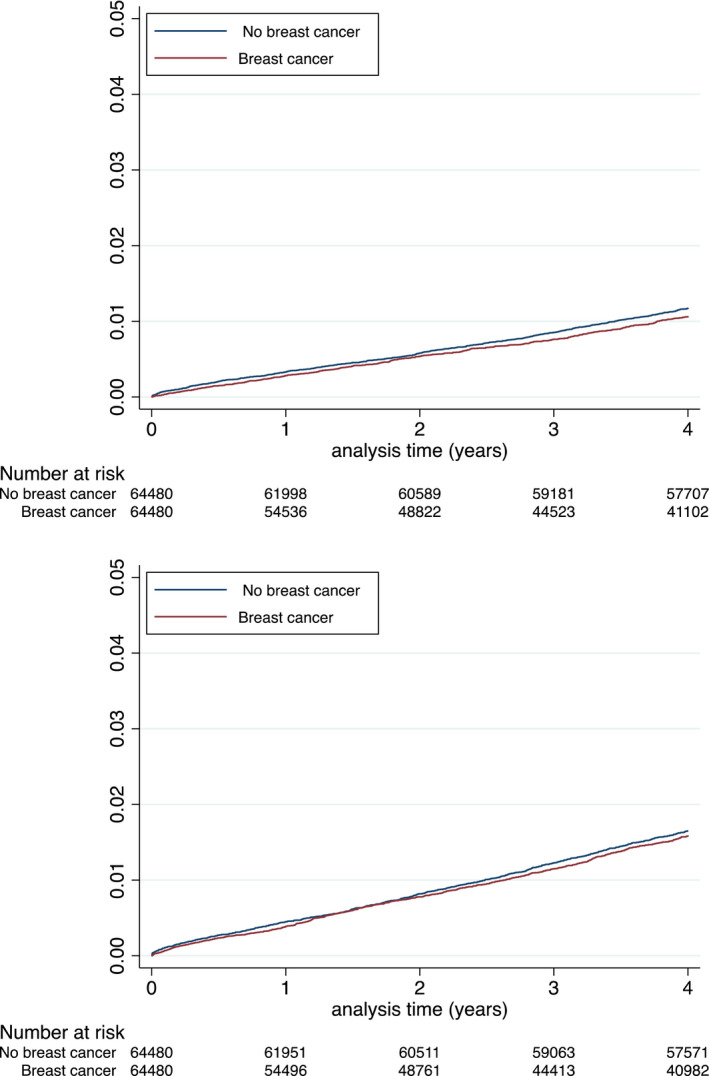
Cumulative incidences for myocardial infarction (top panel) and ischaemic stroke (lower panel) during follow‐up in the matched populations

**FIGURE 4:**
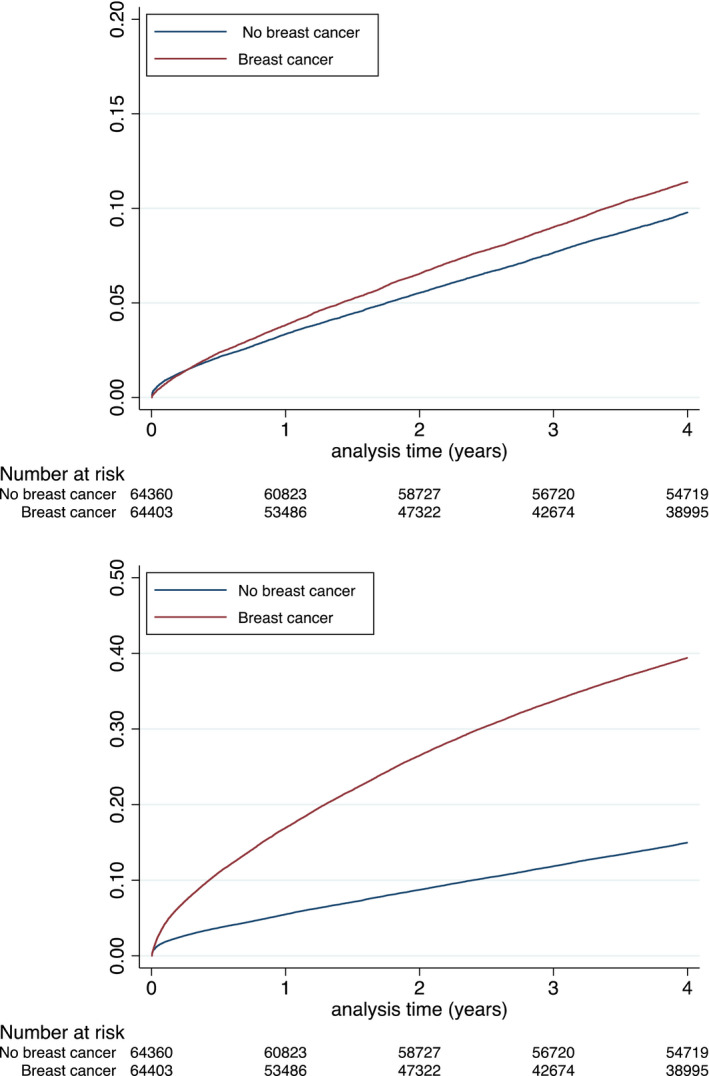
Cumulative incidences for MACE‐HF (top panel) and NACE (lower panel) during follow‐up in the matched populations. MACE‐HF = major cardiovascular events (in‐hospital cardiovascular death, myocardial infarction, ischaemic stroke or new‐onset heart failure). NACE = net adverse clinical events (in‐hospital death, myocardial infarction, ischaemic stroke, new‐onset heart failure or major bleeding)

Using propensity score, 64,480 female patients with history of BC were adequately matched in a 1:1 fashion with female patients with no history of BC patients (Table 2 and Figure S1 and Figure S2). In the matched population, the incidence rates (IRs) of MACE‐HF, cardiovascular death, MI, ischaemic stroke, new‐onset HF and major bleeding are reported in Table 3 (Figures 2, 3, 4). Matched female patients with BC had a higher risk of all‐cause death (HR 3.55, 95% confidence interval [CI]: 3.47–3.64), new‐onset HF (HR 1.08, 95% CI 1.04–1.11), major bleeding (HR 1.43, 95% CI 1.36–1.49), MACE‐HF (HR 1.07, 95% CI 1.04–1.11) and NACE (HR 2.53, 95% CI 2.48–2.58) compared with those with no BC.

**TABLE 2:** Baseline characteristics of matched women seen in French hospitals in 2013 with no history of cancer or history of breast cancer

	No breast cancer	Breast cancer	*p*	Total
	(n = 64480)	(n = 64480)		(n = 128960)
Age, years	63.0 ± 13.7	63.0 ± 13.7	1	63.0 ± 13.7
Sex (male)	0 (0.0)	0 (0.0)	‐	0 (0.0)
Obesity	6479 (10.0)	6484 (10.1)	0.96	12963 (10.1)
Hypertension	17829 (27.7)	17834 (27.7)	0.98	35663 (27.7)
Diabetes mellitus	5866 (9.1)	5873 (9.1)	0.95	11739 (9.1)
Dyslipidaemia	6663 (10.3)	6668 (10.3)	0.96	13331 (10.3)
Smoker	3018 (4.7)	3027 (4.7)	0.91	6045 (4.7)
Alcohol‐related diagnoses	1312 (2.0)	1318 (2.0)	0.91	2630 (2.0)
Valve disease	1117 (1.7)	1032 (1.6)	0.06	2149 (1.7)
Coronary artery disease	2101 (3.3)	1532 (2.4)	<0.0001	3633 (2.8)
Previous PCI	331 (0.5)	165 (0.3)	<0.0001	496 (0.4)
Previous CABG	29 (0.0)	23 (0.0)	0.41	52 (0.0)
Vascular disease	1950 (3.0)	1290 (2.0)	<0.0001	3240 (2.5)
Atrial fibrillation	3017 (4.7)	2586 (4.0)	<0.0001	5603 (4.3)
Previous pacemaker or ICD	751 (1.2)	607 (0.9)	0.0001	1358 (1.1)
Intracranial bleeding	531 (0.8)	316 (0.5)	<0.0001	847 (0.7)
Chronic kidney disease	1213 (1.9)	827 (1.3)	<0.0001	2040 (1.6)
Lung disease	4563 (7.1)	3983 (6.2)	<0.0001	8546 (6.6)
Sleep apnoea syndrome	1862 (2.9)	1115 (1.7)	<0.0001	2977 (2.3)
Liver disease	1371 (2.1)	1424 (2.2)	0.31	2795 (2.2)
Thyroid diseases	5152 (8.0)	5273 (8.2)	0.22	10425 (8.1)
Inflammatory disease	3615 (5.6)	2048 (3.2)	<0.0001	5663 (4.4)
Anaemia	3431 (5.3)	6445 (10.0)	<0.0001	9876 (7.7)
Previous cancer	0 (0.0)	64480 (100.0)	‐	64480 (50.0)
Cognitive impairment	1750 (2.7)	1206 (1.9)	<0.0001	2956 (2.3)
Illicit drug use	70 (0.1)	84 (0.1)	0.26	154 (0.1)

Note

Values are n (%) or mean ± SD.

Mean follow‐up: 4.6 ± 1.8 years; median: 5.4; IQR: 4.6–5.8 years

Abbreviations: CABG, coronary artery bypass graft; ICD, implantable cardioverter–defibrillator; IQR, interquartile range; PCI, percutaneous coronary intervention; SD = standard deviation.

**TABLE 3:** Incident outcomes in the matched population according to breast cancer or no breast cancer

	No breast cancer (n = 64480)		Breast cancer (n = 64480)		*p* value
	Number of events	Incidence, %/yr (95% CI)	Number of events	Incidence, %/yr (95% CI)	
All‐cause death	9803	2.94 (2.88–3.00)	29609	11.22 (11.10–11.35)	<0.0001
Cardiovascular death	2125	0.64 (0.61–0.66)	1742	0.66 (0.63–0.69)	0.26
Noncardiovascular death	7678	2.30 (2.25–2.35)	27867	10.56 (10.44–10.69)	<0.0001
Myocardial infarction	1386	0.42 (0.40–0.44)	938	0.36 (0.34–0.38)	0.0002
Ischaemic stroke	1955	0.59 (0.57–0.62)	1396	0.53 (0.51–0.56)	0.003
New‐onset HF	7794	2.42 (2.37–2.48)	7031	2.76 (2.69–2.82)	<0.0001
MACE‐HF 1	10318	3.24 (3.18–3.30)	9115	3.60 (3.53–3.68)	<0.0001
Major bleeding	3541	1.08 (1.05–1.12)	4187	1.62 (1.57–1.67)	<0.0001
NACE	15562	4.88 (4.81–4.96)	33180	13.12 (12.98–13.26)	<0.0001

Note

MACE‐HF, major cardiovascular events (in‐hospital cardiovascular death, myocardial infarction, ischaemic stroke or new‐onset heart failure).

NACE, net adverse clinical events (in‐hospital death, myocardial infarction, ischaemic stroke, new‐onset heart failure or major bleeding).

By contrast, risks were not higher for cardiovascular death (HR 0.94, 95% CI 0.88–1.00) and were lower for MI (HR 0.81, 95% CI 0.75–0.88) and ischaemic stroke (HR 0.85, 95% CI 0.79–1.11) (Table 4).

**TABLE 4:** Hazard ratio (95% CI) associated with breast cancer (vs no breast cancer) for incident outcomes

	Model A	Model B	Model C	Model D
All‐cause death	3.523 (3.480–3.567)	3.242 (3.202–3.281)	3.336 (3.296–3.378)	3.554 (3.473–3.636)
Cardiovascular death	0.924 (0.881–0.970)	0.942 (0.898–0.988)	0.960 (0.915–1.007)	0.939 (0.881–1.001) *
Noncardiovascular death	4.269 (4.215–4.324)	3.866 (3.817–3.915)	3.988 (3.938–4.040)	4.280 (4.172–4.389)
Myocardial infarction	0.893 (0.837–0.953)	0.816 (0.765–0.871)	0.820 (0.768–0.875)	0.811 (0.746–0.881) †
Ischaemic stroke	0.945 (0.896–0.997)	0.877 (0.831–0.925)	0.880 (0.834–0.929)	0.849 (0.792–0.910) ‡
New‐onset HF	1.154 (1.127–1.182)	1.056 (1.031–1.081)	1.047 (1.022–1.073)	1.079 (1.044–1.114)
MACE‐HF	1.184 (1.160–1.209)	1.037 (1.016–1.059)	1.036 (1.014–1.058)	1.074 (1.044–1.105)
Major bleeding	1.525 (1.478–1.574)	1.330 (1.289–1.372)	1.348 (1.306–1.390)	1.425 (1.362–1.491)
NACE	2.741 (2.710–2.772)	2.412 (2.385–2.440)	2.443 (2.415–2.470)	2.531 (2.483–2.580)

Note

Model A: unadjusted.

Model B: adjusted for age.

Model C: adjusted on all risk factors and noncardiovascular comorbidities from Table 1

Model D: propensity score–matched analysis.

*hazard ratio = 0.757 (0.710–0.807) by the Fine and Gray model for competing risks of cardiovascular and noncardiovascular death. † Hazard ratio = 0.627 (0.577–0.682) by the Fine and Gray model for competing risks of myocardial infarction and all‐cause death. ‡ Hazard ratio = 0.656 (0.612–0.703) by the Fine and Gray model for competing risks of ischaemic stroke and all‐cause death.

MACE‐HF, major cardiovascular events (in‐hospital cardiovascular death, myocardial infarction, ischaemic stroke or new‐onset heart failure).

NACE, net adverse clinical events (in‐hospital death, myocardial infarction, ischaemic stroke, new‐onset heart failure or major bleeding).

## 4 DISCUSSION

In one of the largest observational studies to date of a contemporary cohort of patients with BC, our principal findings are as follows: firstly, patients with BC are at a higher risk of NACE, mainly driven by all‐cause mortality and HF, when compared to a matched cohort of general population. Secondly, although the IR of cardiovascular deaths is not significantly different between the two populations studied, patients with BC have displayed a lower risk of MI and stroke with a much higher risk of HF.

Management strategy for BC commonly comprises cardiotoxic treatments such as mediastinal radiotherapy, anthracycline‐based chemotherapy and hormonal therapy. All these may have implications for cardiovascular outcomes, but the data are conflicting. In our contemporary cohort, conventional cardiovascular risk factors were comparable between the 2 cohorts despite patients with BC being on average older. BC patients are more likely to be hypertensive patients, dyslipidaemic patients and smokers, whereas they were less likely to be diabetics, with previous coronary artery disease or vascular disease.

Importantly, we have shown that although with advances in the treatment of BC, increase in the survival rates and an overall trend of decreasing mortality rates through the years,^21^ their overall mortality remains approximately 3.5‐fold higher than the general population. There does not appear to be any difference in the cardiovascular mortality in the 2 different groups underlining the noncardiovascular implications of BC. As the mean follow‐up period of our population was 5.1 years, it is in keeping with a previous report by Afifi et al. where the greatest proportion of mortality was within 1 to 5 years related to BC itself even though heart disease remained the most common noncancer mortality.^22^ Cardiovascular mortality surpasses other causes of mortality >10 years after diagnosis.^22^ This highlights the need firstly to further improve noncardiovascular outcomes in this cohort of patients in the early post‐diagnosis period and secondly to give more (and early) focus on cardiovascular risk prevention strategies to improve their long‐term cardiovascular outcomes.

As shown in Table 3, patients with BC, when compared to a matched cohort of patients without BC, are at a higher risk of all‐cause death, new‐onset HF, major bleeding, MACE‐HF and NACE. The increase in MACE‐HF was mainly driven by new‐onset HF in BC patients, and NACE was driven by the combination of a much higher all‐cause death and new‐onset HF. This does not come as a surprise as the main chemotherapeutic agent used in the treatment of BC is anthracycline‐based, and its use has been previously linked to an increased risk of drug‐induced congestive heart failure,^23^, ^24^ which is the most likely explanation for the observation within our study.

Interestingly, there was no corresponding increase in risk of cardiovascular death but instead a reduction in the risk of MI and stroke, which most likely balances out the cardiovascular deaths from the increased incidence of HF. The reduction in MI and stroke is noteworthy as there have been previous suggestions that tamoxifen, a commonly used adjuvant endocrine therapy, although inconclusive (relative risks: 0.91, 95% CI: 0.77–1.07), may have cardioprotective effects in cancer patients.^25^, ^26^ Tamoxifen is an effective antioxidant and protects low‐density lipoprotein particles against oxidative damage^27^ and in combination with its anti‐inflammatory properties has been thought to reduce risk of cardiovascular events, in particular MI.^28^ This could potentially explain the lower risks of MI and stroke seen in our population. Unfortunately, as we did not have access to the detailed drug therapies that the patients were taking, further subanalyses to test this hypothesis could not be performed.

On the contrary, a recent study with follow‐up data of 25 years has shown that BC survivors had an increase in CVD‐related deaths beginning at 8 years after their diagnosis.^29^ Similar to our study, Ramin et al. were unable to conduct subgroup analyses based on treatment.^29^ Therefore, it is possible that the median follow‐up of 5.1 years is insufficient to look into the long‐term CV impact on BC survivors, and with longer follow‐up, the risk of CVD‐related deaths in our cohort may be significantly higher.

### 4.1 Limitations

Firstly, being a retrospective database analysis, potential unknown confounders could have had an impact on the conclusions generated. However, as the results are largely in keeping with a more contemporary cohort,^16^ we believe that the conclusions drawn are reliable. Secondly, despite our best effort in propensity matching, the cohort remains dissimilar in terms of some diseases, which could impact on the cardiovascular outcomes, namely known coronary and vascular disease. Lastly, as we did not have access to the medication records or the disease staging of the BC patients, we were unable to perform further subgroup analyses exploring the impact of the various chemotherapeutic agents and disease subtypes, which would likely explain the higher incidence but lower incidence rates of HF in the non‐BC group when compared to the BC group despite similar follow‐up periods (non‐BC vs BC mean follow‐up of 5 ± 1.5 vs 4 ± 2.1 years).

## 5 CONCLUSIONS

In a large and contemporary analysis of female patients seen in French hospitals, women with history of breast cancer have a higher risk of all‐cause mortality, new‐onset heart failure and major bleeding compared to a matched cohort of women without breast cancer. In contrast, they have a reduced risk of cardiovascular mortality, MI and stroke. Further studies are needed to investigate both the cardiotoxic and cardioprotective impact of the chemotherapeutic agents and how they could be best used to improve outcomes in this high‐risk cohort of patients.

## CONFLICT OF INTEREST

GYHL is a consultant and speaker for BMS/Pfizer, Boehringer Ingelheim and Daiichi‐Sankyo. No fees are received personally. LF reports personal fees from Bayer, personal fees from BMS Pfizer, personal fees from Boehringer Ingelheim, personal fees from Medtronic and personal fees from Novartis, outside the submitted work. Other authors have no conflict of interests to disclose.

## AUTHOR CONTRIBUTION

YXG wrote the first draft and worked on subsequent revisions. ABi, ABo and JH involved in the concept, design and data collection/acquisition and analysis. GYHL was responsible for the conception and design, critical review and revision of the manuscript. LF was responsible for the conception and design, data analysis, critical review and revision of the manuscript.

## Supporting information

Supplementary Material
