# Supplementary material for: Breast cancer and incident cardiovascular events: A systematic analysis at the nationwide level
Source: Eur J Clin Invest. 2022 Feb 10;52(6):e13754. doi: 10.1111/eci.13754 (PMC9285743; doi:10.1111/eci.13754)
Supplement: Supplementary file 1 — Supplementary Material [file ECI-52-0-s001.docx]

**Supplemental figure 1.** Standardized percentages of bias across main baseline characteristics in unmatched and matched patients with breast cancer and no breast cancer.

**Supplemental figure 2.** Propensity score distribution for unmatched and matched populations of patients with breast cancer and no breast cancer.
